# Lateral tibiofemoral morphometry does not identify risk of re-ruptures after ACL reconstruction in children and adolescents

**DOI:** 10.1186/s40634-021-00403-5

**Published:** 2021-10-08

**Authors:** Martijn Dietvorst, Stéphanie Verhagen, M. C. (Marieke) van der Steen, Peter Faunø, Rob P. A. Janssen

**Affiliations:** 1 Department of Orthopaedic Surgery & Trauma, Máxima MC, Eindhoven, the Netherlands; 2 Department of Orthopaedic Surgery & Trauma, Catharina Hospital Eindhoven, Eindhoven, the Netherlands; 3 Department of Orthopaedic Surgery, Aarhus University Hospital, Aarhus, Denmark; 4 Orthopaedic Biomechanics, Department of Biomedical Engineering, Eindhoven University of Technology, Eindhoven, the Netherlands; 5 Chair Value-Based Health Care, Department of Paramedical Sciences, Fontys University of Applied Sciences, Eindhoven, the Netherlands

## Background

As the age of onset for anterior cruciate ligament (ACL) ruptures in children decreases, the incidence of ACL ruptures in children increases [1, 34]. ACL injuries are a severe injury to the paediatric knee [2]. A primary concern after ACL reconstruction in children and adolescents is the increased risk of ipsilateral graft ruptures and contralateral ACL ruptures [2, 9, 19, 21, 34]. It is therefore important to be aware of potential morphological risk factors in light of other intrinsic and extrinsic risk factors, such as an increased body mass index (BMI) and sports participation, that can potentially identify children prone for re-ruptures [23].

Previous studies reported several morphological risk factors for primary ACL ruptures in children and adolescents [6–8, 22, 32]. Risk factors in children include an increased medial and lateral tibial slope, narrower notch width, increased size of tibial eminence and patella alta [6–8, 22, 32]. The tibial slope has been studied previously as a morphological risk factor for re-ruptures or revisions in children and adolescents [5, 12, 26].

Recently, the morphology of the lateral compartment of the knee gained more interest in relation to ACL (re-)ruptures in adults, as it may play an important role in the pivot shift phenomenon and knee kinematics [10, 14, 24, 27, 31]. The lateral tibial slope and meniscal bone angle have been identified as risk factors for re-ruptures in adults [4, 13, 16, 27]. Studies on the lateral femoral condyle shape were somewhat contradictory [14, 17, 24]. Pfeiffer et al. [24] showed that an increased posterior femoral depth, defined as an increased femoral condyle ratio, is associated with an increased risk of primary and contralateral ACL injuries [24]. This might be due to a greater anisometry in flexion because of increased length of anterolateral and lateral structures, resulting in laxity near full extension, which is the point where most non-contact ACL injuries occur [24, 25]. Hodel et al. [14] however, showed that a decreased lateral femoral condyle index (LFCI), resulting in a more spherical shape of the femur, is associated with an increased risk of primary ACL injuries [14]. A decreased lateral femoral condyle index consists of a smaller posterior flexion circle and therefore a more prominent anterior part of the condyle, resulting in excessive gliding of the flat surface of the condyle over the convex tibial plateau [14]. Besides, patients with a decreased LFCI in combination with an increased lateral tibial slope and lateral tibial height are at higher risk for an ACL rupture and re-rupture [14]. Another study on tibiofemoral morphometric relations reported that an increased Porto ratio is a risk factor for primary ACL injury [10]. These results might suggest that bony morphology of both the femoral condyle and tibial plateau play an important role in knee kinematics and the Pivot-Shift magnitude and risk of ACL injuries [10, 14, 18].

The aim of this study was to evaluate the tibiofemoral morphology of the lateral knee compartment on magnetic resonance imaging (MRI) as risk factors for ipsilateral graft rupture and contralateral ACL rupture after ACL reconstruction in children and adolescents in a case control study. The hypothesis was that an increased lateral tibial slope, a decreased femoral flat surface, a decreased meniscal bone angle and a decreased lateral femoral condyle index were morphological risk factors for ipsilateral graft ruptures and contralateral ACL ruptures in children and adolescents after ACL reconstruction.

## Methods

### Patients

The study design was a case control study and approved by both the local medical ethical committee Máxima Medical Centre [L19.047] and Aarhus University Hospital. A retrospective chart review was conducted for children and adolescents (< 18 years old) who underwent primary ACL reconstruction at the Máxima MC Eindhoven, The Netherlands and Aarhus University Hospital, Denmark. Inclusion and exclusion criteria are shown in Table 1.

**Table 1:** In- and exclusion criteria for cases and controls

Inclusion criteria		Exclusion criteria
Cases	Controls	Overall
Age < 18 years^a^	Age < 18 years^a^	Absence of preoperative MRI or impossibility to perform measurements on the MRI due to absence of slices or insufficient quality
Primary ACL reconstruction	Primary ACL reconstruction	Absence of preoperative information required for matching
Re-rupture: Ipsilateral graft rupture or contralateral ACL rupture	No ipsi- or contralateral ACL injury	Revision due to infection
	Minimum follow-up of 1 year	

^a^ < 18 years at time of primary ACL reconstruction

The cases were matched to the controls according to gender, age, height, weight and surgical technique. Matching on surgical technique consisted of adult surgical technique versus surgical technique for open physes (all-epiphyseal, transphyseal, hybrid), graft use and concomitant anterolateral ligament (ALL) reconstruction. Control patients of the MMC were contacted by telephone to reassure there was no re-rupture or contralateral ACL injury. Control patients of AUH were not contacted, due to the organisation of the electronic patient files which include all hospitals in Jutland, Denmark. Consultations in other hospitals for re-rupture or contralateral ACL injury could therefore be found in the electronic patient files. After matching, the study consisted of two separate populations: (1) the ipsilateral ACL graft rupture cases and their matched controls, and (2) the contralateral ACL rupture cases and their matched controls.

**Fig. 1:**
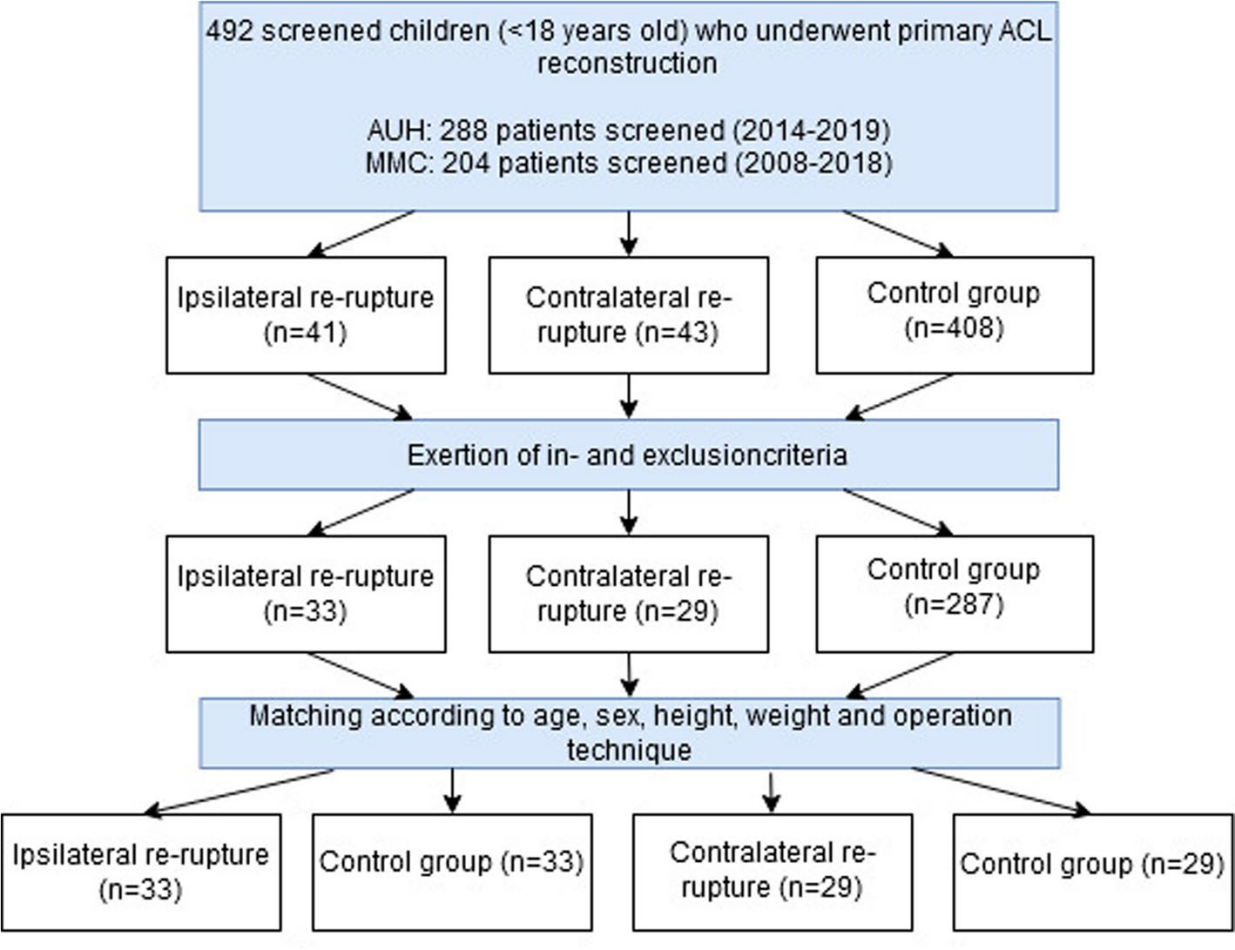
Flow chart of patient in- and exclusion; “ACL = Anterior Cruciate Ligament”, “AUH = Aarhus University Hospital”, “MMC = Máxima Medical Centre”

A total of 492 medical files of children and adolescents after primary ACL reconstruction were screened. After exertion of the in- and exclusion criteria, 33 patients were included for having an ipsilateral graft re-rupture, and 29 patients for having a contralateral ACL rupture (Fig. 1). Patients screened for the re-rupture groups who did not meet the inclusion criteria were most often excluded because there was no pre-operative MRI available. Patients in the control group who did not meet the inclusion criteria usually lacked a follow-up period of 12 months or in some cases the pre-operative MRI was unavailable.

### MRI measurements

As many patients were secondary referrals to our clinics, preoperative MRI’s of the primary ACL rupture were performed with many different types of MRI’s.

The measurements regarding morphologic characteristics were performed using the preoperative MRI’s of the primary ACL rupture. MRI’s were imported in Agfa Healthcare IMPAX version 6.6.1.3004 (Agfa HealthCare®, Mortsel, Belgium) to perform the measurements. Measurements were performed on sagittal images on the Proton Density Weighted (PDW) series. Coronal series were necessary to determine the positioning of the correct slice for the measurements of the lateral compartment.

#### Tibia

**Fig. 2:**
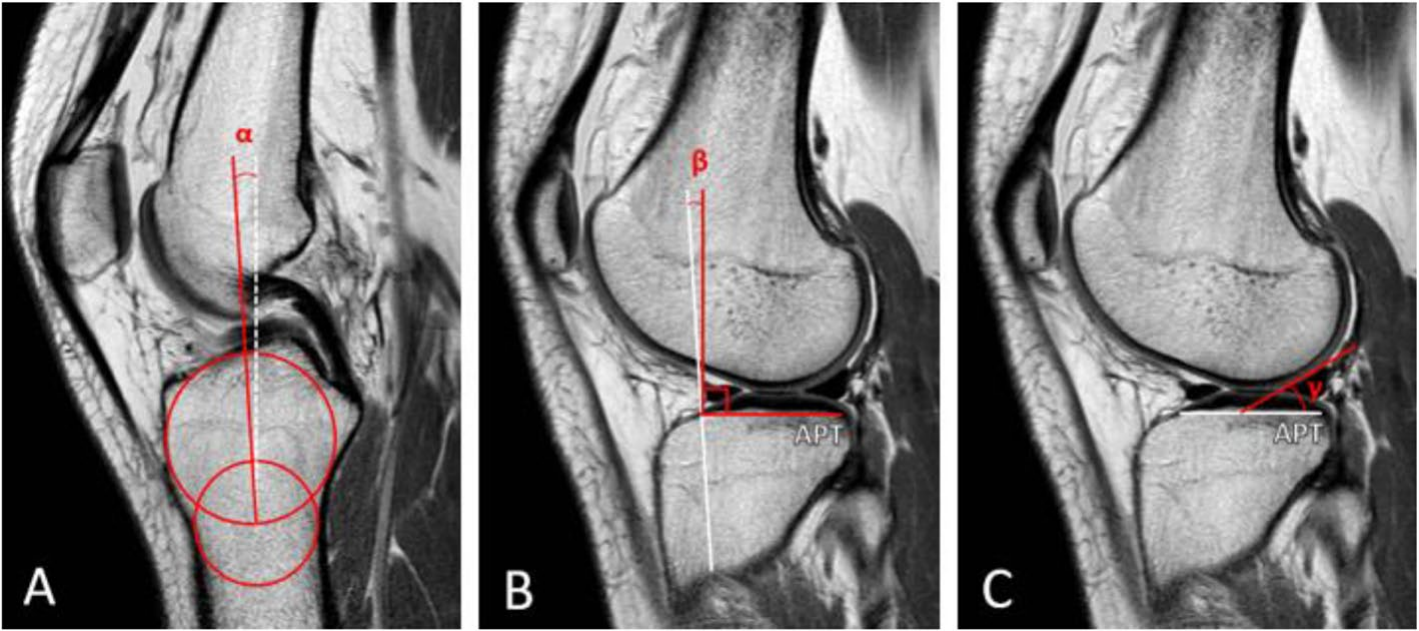
MRI measurements of the tibia. **A** The longitudinal tibial axis (α) is drawn. **B** After drawing the AP depth of the tibial plateau [APT], α is used to determine the tibial slope (β). **C** The meniscal bone angle (γ) is drawn using the APT

The tibial parameters of interest were the tibial slope, the meniscal bone angle and the anterior-posterior depth of the tibial plateau (Fig. 2). First the tibial axis was measured by the method of Hudek [15]. Then, the centre of the lateral compartment was determined based on the AP slices as stated by Hodel et al. [14] Measurements of the tibial slope were performed following the method by Hodel et al. [14], the meniscal bone angle by Sauer et al. [27, 30], the AP depth of the tibia by Shaw et al. [28]

#### Femur

**Fig. 3:**
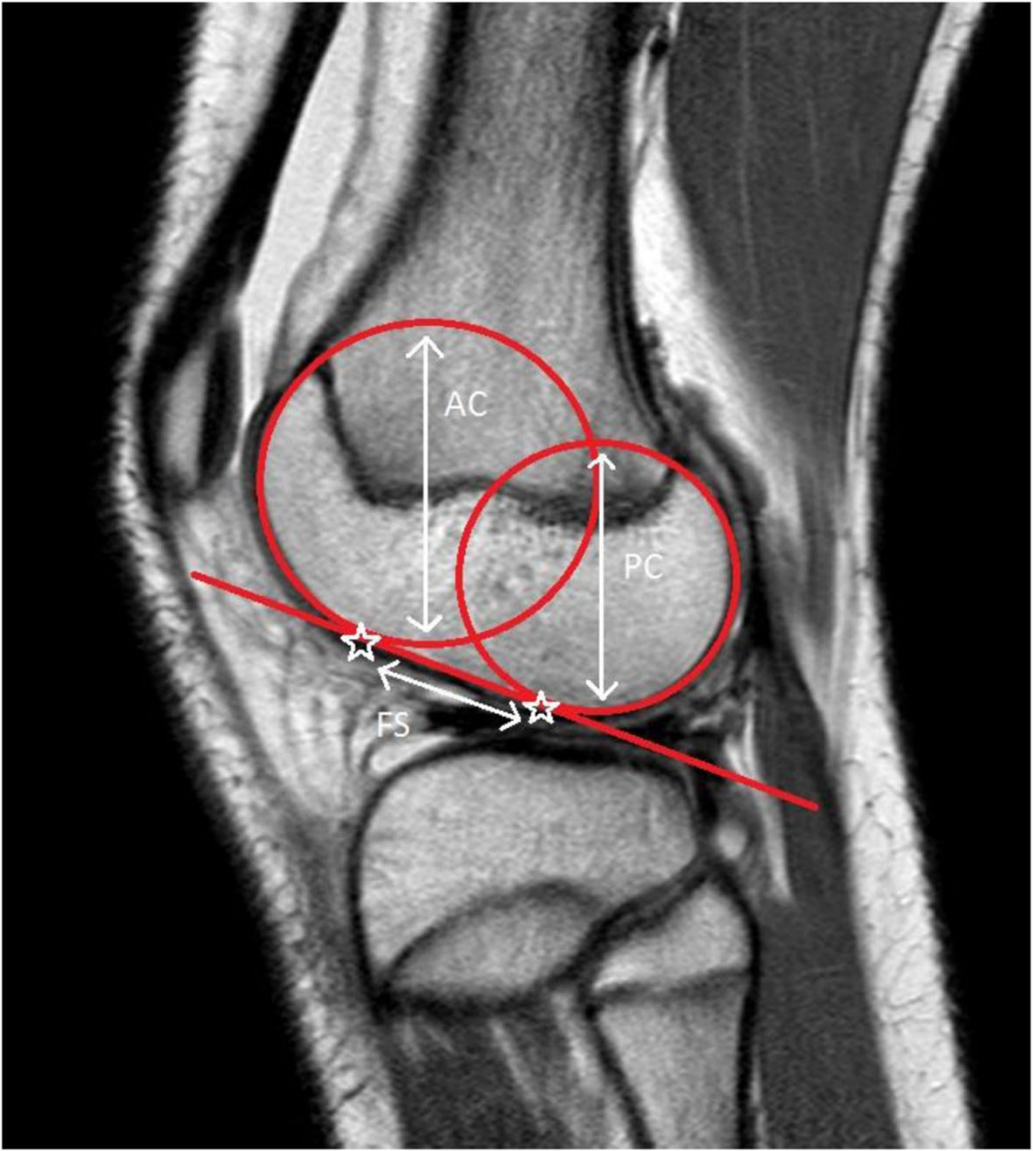
MRI measurements of the femur. The anterior “extension” circle [AC] and posterior “flexion” circle [PC] are drawn [14]. A line tangent to both circles determines the flat surface [FS] [31].

The measurements of the femur were performed on the same slice as the measurements on the tibia. Parameters of interest of the femur were the diameter of the anterior extension circle, posterior flexion circle and the flat surface (Fig. 3). The measurements of the circles were performed following the methods by Hodel et al. [14] The flat surface was measured according to the method by Vasta et al. [31]

#### Indices

The lateral femoral condyle index (LFCI) is calculated by dividing the diameter of the posterior flexion circle by the diameter of the anterior extension circle (Fig. 3) [14]. Second, the Porto ratio is calculated by dividing the flat femur surface (Fig. 3) by the AP depth of the tibial plateau (Fig. 2B) [31].

### Inter- and intra-observer reliability

To determine the inter- and intra-observer reliability, all measurements were performed on twenty randomly selected MRI’s from the pre-matched research population. Two blinded reviewers (M.D., S.V.) performed the measurements independently to determine the inter-observer reliability. Both of the reviewers repeated all twenty measurements 1 week later to determine the intra-observer reliability.

### Statistical analysis

The inter- and intra-observer reliability were determined by calculating the Intraclass Correlation Coefficient (ICC) with a 95%-Confidence Interval (CI).

Normality of continuous data was tested using with Shapiro Wilk test. Differences in baseline characteristics between the ipsilateral and contralateral re-injured patients were tested by means of Mann-Whitney U or unpaired t-tests.

A sample size calculation was based on outcomes of the lateral tibial slope in the study by Jaecker et al. [16] (mean (SD) lateral tibial slope in graft failure patients 7.3° (3.3) versus controls 4.5° (3.2)), as the tibial slope was determined as the potential most important risk factor. A paired t-test sample size estimation in G*Power resulted in a group size of 26 patients (correlation of 0.01, α of 0.05 and power of 0.85).

All statistical analyses on morphological risk factors were performed both separately for the ipsilateral graft rupture and contralateral ACL rupture group and their matched control group, as well as for the total ACL re-rupture group and the total group of matched controls. The data within these analyses was approached as paired as a results of matching. Depending of distribution of continuous data the paired t-test or Wilcoxon signed rank test was used. Categorical data were analysed by means of Wilcoxon signed rank test or McNemar test.

For each parameter, the receiver operating characteristic (ROC) curve, the area under the curve (AUC) and its 95% CI were calculated. The AUC represents the diagnostic accuracy of the measurements, ranging from 0 to 1.0 (perfect test), with 0.5 as cut-off for no discrimination capacity. A 2-sided binomial z test was performed to determine the statistical significance of the AUC. Statistical analyses were computed using SPSS version 22. The significance level was set at 0.05.

## Results

### Inter- and intra-observer reliability

All measurements showed good to excellent inter- and intra-observer reliability (Table 2).

**Table 2:** Inter- and intra-observer reliability of morphological parameters ^a^

	Inter-observer reliability	Intra-observer reliability	
		(M.D.)	(S.V)
Lateral tibial slope	0.93 (0.83–0.97)	0.90 (0.75–0.96)	0.90 (0.75–0.96)
Meniscal bone angle	0.90 (0.75–0.96)	0.95 (0.87–0.98)	0.95 (0.88–0.98)
AP tibia depth	0.99 (0.63–0.94)	0.96 (0.90–0.99)	0.97 (0.93–0.99)
Femur flat surface	0.90 (0.74–0.96)	0.88 (0.69–0.95)	0.92 (0.79–0.97)
Lateral femoral condyle index	0.77 (0.42–0.91)	0.77 (0.42–0.91)	0.76 (0.39–0.91)
Porto ratio	0.86 (0.66–0.95)	0.89 (0.72–0.96)	0.91 (0.78–0.97)

^a^Values are presented as intraclass correlation coefficient (95% CI). *AP* Anterior-posterior, *CI* Confidence interval

### Patients

The demographic characteristics of the ipsilateral and contralateral matches are shown in Table 3. There were no differences in the baseline characteristics between the re-injured patients and control patients for both groups, indicating a successful matching process. There were no differences in age, height, weight and BMI between the ipsilateral and contralateral re-injured patients. There were, however, significantly more males in the ipsilateral graft rupture group and more females in the contralateral ACL group (respectively 70% and 66%, *p* = 0.01). There were no statistically significant differences in the time to re-rupture and surgical technique between the ipsi- and contralateral injured patients.

**Table 3:** Demographic characteristics ipsi- and contralateral matches

	Ipsilateral			Contralateral		
	Injured (33)	Control (33)	***P***-value	Injured (29)	Control (29)	***P***-value
Age, years (mean SD)	14.5 (1.7)	14.5 (1.8)	0.414	14.9 (2.0)	15.1 (1.4)	0.180
Gender, n female (%)	10 (30)	10 (30)	1.000	19 (66)	19 (66)	1.000
Weight, kg (mean SD)	63 (18)	61 (13)	0.401	62 (11)	64 (9.7)	0.085
Height, cm (mean SD)	171 (12)	171 (12)	0.760	172 (9.0)	171 (9.3)	0.635
BMI, kg/m^2^ (mean SD)	21 (4.2)	21 (2.7)	0.438	21 (3.1)	22 (2.2)	0.094
Time to rerupture			n.a.			n.a.
< 1 year	5	n.a.		1	n.a.	
1–2 years	12	n.a.		15	n.a.	
> 2 years	16	n.a.		12	n.a.	
Follow-up duration controls, years (mean SD)	n.a.	4.3 (2.3)	n.a.	n.a.	4.2 (2.3)	n.a.
Surgical technique			0.059			0.705
Adult	17	16		21	22	
Hybrid	12	16		5	6	
Transphyseal	4	1		2	1	
All-epiphyseal	0	0		1	0	
Graft type			0.317			1.000
HS	32	33		29	29	
BPTB	1	0		0	0	
QT	0	0		0	0	
Allograft	0	0		0	0	
Primary ALL reconstruction			1.000			1.000
Yes	1	0		1	2	
No	32	33		28	27	

*ALL* Anterolateral ligament, *BPTB* Bone patella tendon bone, *cm* Centimetres, *HS* Hamstring, *kg* Kilograms, *LCL* Lateral collateral ligament, *m* Metre, *MCL* Medial collateral ligament, *n.a.* Not applicable, *PCL* Posterior cruciate ligament, *QT* Quadriceps tendon, *SD* Standard deviation

### Morphological risk factors

There were no statistically significant differences between the measurement of the lateral side of the knee in both the ipsilateral and the contralateral matches, as shown in Table 4. Analysis of the total group of re-ruptures (ipsilateral graft and contralateral ACL) and their matches showed that the re-injured patients had a significant greater tibial slope (*p* = 0.048). Analyses of the degree of tibial slope and the association with re-injuries showed that a tibial slope ≥ 7˚ was associated with re-injuries, as significantly more patients with a slope of ≥ 7˚ had re-injuries (*p*=0.023).

**Table 4:** MRI parameters in the ipsilateral and contralateral group, shown as median [IQR]

	Ipsilateral			Contralateral			Total		
	Re-injured (33)	Controls (33)	***P***-value	Re-injured (29)	Controls (29)	***P***-value	Re-injured (62)	Controls (62)	***P***-value
Tibial slope (^0^)	5 [2–8]	3 [2–6]	0.233	5 [4–7]	5 [3–6]	0.104	5 [3–8]	4 [2–6]	0.048
Meniscal bone angle (^0^)	24 [22–28]	25 [22–29]	0.941	27 [23–30]	27 [24–30]	0.739	26 [23–29]	26 [23–29]	0.779
AP depth tibia (mm)	30 [29–37]	32 [29–35]	0.821	32 [30–36]	33 [30–35]	0.968	32 [29–36]	32 [30–35]	0.913
Flat surface femur (mm)	21 [18–25]	19 [16–25]	0.215	25 [19–27]	25 [19–28]	0.909	24 [18–26]	22 [18–27]	0.401
LFCI	0.75 [0.66–0.81]	0.72 [0.67–0.76]	0.257	0.71 [0.67–0.75]	0.71 [0.66–0.75]	0.907	0.73 [0.67–0.79]	0.71 [0.66–0.76]	0.302
Porto ratio	0.66 [0.55–0.77]	0.61 [0.48–0.73]	0.397	0.79 [0.66–0.84]	0.76 [0.61–0.84]	0.899	0.74 [0.60–0.82]	0.69 [0.57–0.80]	0.431

*AP* Anterior-posterior, *IQR* Interquartile range, *LFCI* Lateral femoral condyle index, *mm* Millimetres, *MRI* Magnetic resonance imaging

### Diagnostic performance of MRI measurements

In Table 5, the AUC’s for each measurement is shown. None of the AUC showed significant difference from 0.5.

**Table 5:** Area under the curve (AUC) for each parameter in the ipsi- and contralateral and total group

	Ipsilateral		Contralateral		Total	
	AUC	95%-CI	AUC	95%-CI	AUC	95%-CI
Tibial slope	0.578	0.436–0.719	0.590	0.442–0.738	0.589	0.488–0.690
Meniscal bone angle	0.495	0.353–0.637	0.477	0.327–0.627	0.485	0.382–0.587
AP tibia depth	0.507	0.364–0.650	0.520	0.369–0.671	0.502	0.399–0.605
Flat surface femur	0.566	0.426–0.707	0.475	0.325–0.625	0.519	0.416–0.621
Lateral femur condyle index	0.588	0.449–0.728	0.507	0.356–0.657	0.549	0.447–0.651
Porto ratio	0.574	0.434–0.714	0.529	0.378–0.680	0.545	0.443–0.647

*AP* Anterior-posterior, *AUC* Area under the curve, *CI* Confidence interval

## Discussion

The most important finding of the present study is that the morphological measurements of the lateral compartment of the knee are not found to be statistically significant risk factors for ipsilateral graft ruptures and contralateral ACL ruptures in children and adolescents. The total re-injured population had a significant greater lateral tibial slope compared to controls and tibial slopes of ≥ 7˚ were associated with re-injuries. Tibial slope was however not identified as discriminitive factor for identifying the risk of re-injuries.

The only morphological measurement of the lateral compartment of the knee previously investigated as a risk factor for re-ruptures in adolescents is the tibial slope [5, 12, 26]. Outcomes of these studies showed different results [5, 12, 26]. In contrast to the current study, two studies previously reported tibial slope as a risk factor for re-ruptures after ACL reconstruction [12, 26]. Salmon et al. [26] found that the sagittal tibial slope on radiographs is a strong predictor for ipsilateral graft ruptures and contralateral ACL injury after reconstruction and has even more negative effects in adolescents compared to adults [26]. Grassi et al. [12] found that a steep medial tibial plateau slope is associated with a higher risk of contralateral ACL injury within 2 years after ACL reconstruction [12]. Cooper et al. [5] found no association between revision of ACL and medial and lateral posterior tibial slope, although extreme measurements of the lateral posterior tibial slopes were associated with ACL revision surgery [5]. As in the study bij Cooper et al., in the current study higher degrees of tibial slope were associated with re-injuries [5]. Differences in outcomes between studies should be interpreted in light of the multifactorial causes for ACL (re-)ruptures, as besides to morphological factors, other intrinsic and extrinsic factors are known to be risk factors for ACL (re-)injury [11, 23]. Caution should also be taken when comparing outcomes from different studies, due to various measurement methods, alternative case definitions (graft failure versus revision) and variations in follow-up and return to sports timing [5, 31].

The role of several other morphological measurement of the lateral compartment of the knee as risk-factors for re-ruptures had been investigated in adults. Differences in the current study focussing on adolescent compared to the in literature known outcomes in adults were found. Previous studies reported the lateral tibial slope as a risk factor for re-ruptures after ACL reconstruction in adults, which is in contrast to the outcomes of the current study [4, 13, 16]. A decreased meniscal bone angle was associated with ipsilateral re-ruptures after ACL reconstruction in adults [27]. However, in the current study, there was no difference in the meniscal bone angle between re-injured and control patients. Variations in the shape of the femoral condyle were not associated with a risk of re-ruptures, which is in accordance with the study by Hodel et al. [14], but in contrast to the study by Pfeiffer et al. [24] In the study by Hodel et al. [14], a decreased LFCI measured on MRI, resulting in a more spherical condyle, was associated with higher risk on primary ACL ruptures compared to controls [14]. The LFCI was however not different between patients with re-ruptures and no re-ruptures after ACL reconstruction [14]. In the study by Pfeiffer et al. [24], the lateral femoral condyle ratio (LFCR), measured on radiographs, was significantly higher in patients with contralateral re-ruptures compared to patients without re-ruptures after ACL reconstruction [24]. Interestingly, Hodel et al. [14] reported that patients with a decreased LFCI in combination with an increased lateral tibial slope and lateral tibial height are at higher risk for an ACL rupture and re-rupture [14]. A specific tibiofemoral morphological outcome, the Porto ratio, was previously investigated as a risk factor for primary ACL injuries, but not as a risk factor for re-ruptures [10]. In the current study, the Porto ratio was also not identified as a risk factor for re-ruptures. Comparing these study outcomes in adults with outcomes in children and adolescents is difficult due to different intrinsic and extrinsic factors. With regard to the risk of re-ruptures, it is known that children and adolescents have an increased risk compared to adults [2, 3, 9]. Furthermore, it is known that certain morphological parameters might change during growth, such as the medial and lateral tibial slope [6]. In contrast with adults, not morphological parameters but rather other factors associated with multifactorial nature of the risk for re-ruptures might play a more prone role in adolescents [23].

Certain demographic differences were found in this study between females and males. The ipsilateral graft rupture group consisted of 70% males, the contralateral ACL rupture group 66% females. The percentage of males with ipsilateral graft ruptures is in accordance with the study by Astur et al. [3] Salmon et al. [26] also showed that adolescent males have an increased risk of graft failure, compared to adults and females [26]. Previous reports on gender distribution in contralateral ACL ruptures show variable results [11, 20, 29, 33].

An important limitation of this study is the inability to investigate other possible relevant parameters, such as the intercondylar notch width. The intercondylar notch was found to be a statistically significant risk factor for primary ACL rupture in children [8]. The current study did not include this parameter, as a notch plasty is performed during ACL reconstructions in some patients to prevent graft failure. The preoperative notch might therefore have different characteristics compared to the postoperative notch. It seemed therefore inappropriate to investigate the preoperative notch shape as a risk factor for graft ruptures.

Compared to previous studies on this topic, the current study also had several strengths. The current study has a relatively large population of re-ruptures due to the combined analyses of the patients of Aarhus University Hospital and Máxima MC, both PAMI (Paediatric ACL Monitoring Initiative) participating centres [21]. Furthermore, the study population also contained a relatively large number of skeletally immature children with an ACL reconstruction technique for open physes. Other studies of morphological risk factors on re-ruptures focussed on the tibial plateau, as the current study intended to evaluate tibiofemoral morphological relations [5, 12, 26]. Another methodical strength is that measurements were performed on MRI. As stated by Dare et al. [6], measurements of bony morphology in children on MRI is superior to standard radiographs, as the subchondral bone in skeletally immature children do not adequately represent the articular surface [6].

The clinical relevance of this study is that the investigated morphological parameters of the lateral compartment of the knee were not found to be significant risk factors for ipsilateral graft ruptures and contralateral ACL ruptures after ACL reconstruction in children and adolescents. Although not discriminitive for identifying re-injuries, tibial slopes ≥ 7˚ were associated with re-injuries. Future studies should focus on morphological risk factors in a more multifactorial role of intrinsic and extrinsic factors, including also postoperative rehabilitation and type of sports participation.

## Conclusion

The investigated morphological parameters of the lateral compartment of the knee were not found to be significant risk factors for ipsilateral graft ruptures and contralateral ACL ruptures in children and adolescents. The total re-injured population had a significant greater lateral tibial slope compared to controls and slopes ≥ 7˚ were associated with re-injuries. The lateral tibial slope was however not a discriminative factor for identifying risk of re-ruptures.

## Abbreviations

ACL: Anterior Cruciate Ligament
ALL: AnteroLateral Ligament
AP: Anterior-Posterior
AUC: Area Under the Curve
AUH: Aarhus University Hospital
BPTB: Bone Patella Tendon Bone
CI: Confidence Interval
HS: Hamstrings
ICC: Intraclass Correlation Coefficient
IQR: Interquartile Range
LCL: Lateral Collateral Ligament
LFCI: Lateral Femoral Condyle Index
LFCR: Lateral Femoral Condyle Ratio
MCL: Medial Collateral Ligament
Máxima MC: Máxima Medical Centre
MRI: Magnetic Resonance Imaging
n.a.: Not applicable
PAMI: Paediatric ACL Monitoring Initiative
PCL: Posterior Cruciate Ligament
QT: Quadriceps Tendon
ROC: Receiving Operating Characteristics
SD: Standard Deviation

## References

[1] Accadbled F, Gracia G, Laumonerie P, Thevenin-Lemoine C, Heyworth BE, Kocher MS (2019). Paediatric anterior cruciate ligament tears: management and growth disturbances. A survey of EPOS and POSNA membership. J Child Orthop.

[2] Ardern CL, Ekås GR, Grindem H, Moksnes H, Anderson AF, Chotel F (2018). 2018 International Olympic Committee consensus statement on prevention, diagnosis and management of paediatric anterior cruciate ligament (ACL) injuries. Br J Sports Med.

[3] Astur DC, Novaretti JV, Cavalcante ELB, Goes A, Kaleka CC, Debieux P (2019). Pediatric anterior cruciate ligament Reruptures are related to lower functional scores at the time of return to activity: a prospective, midterm follow-up study. Orthop J Sports Med.

[4] Christensen JJ, Krych AJ, Engasser WM, Vanhees MK, Collins MS, Dahm DL (2015). Lateral tibial posterior slope is increased in patients with early graft failure after anterior cruciate ligament reconstruction. Am J Sports Med.

[5] Cooper JD, Wang W, Prentice HA, Funahashi TT, Maletis GB (2019). The association between Tibial slope and revision anterior cruciate ligament reconstruction in patients ≤21 years old: a matched case-control study including 317 revisions. Am J Sports Med.

[6] Dare DM, Fabricant PD, McCarthy MM, Rebolledo BJ, Green DW, Cordasco FA (2015). Increased lateral tibial slope is a risk factor for pediatric anterior cruciate ligament injury: an MRI-based case-control study of 152 patients. Am J Sports Med.

[7] Degnan AJ, Maldjian C, Adam RJ, Fu FH, Di Domenica M (2015). Comparison of Insall-Salvati ratios in children with an acute anterior cruciate ligament tear and a matched control population. AJR Am J Roentgenol.

[8] Domzalski M, Grzelak P, Gabos P (2010). Risk factors for anterior cruciate ligament injury in skeletally immature patients: analysis of intercondylar notch width using magnetic resonance imaging. Int Orthop.

[9] Faunø P, Rahr-Wagner L, Lind M (2014). Risk for revision after anterior cruciate ligament reconstruction is higher among adolescents: results from the Danish registry of knee ligament reconstruction. Orthop J Sports Med.

[10] Fernandes MS, Pereira R, Andrade R, Vasta S, Pereira H, Pinheiro JP (2017). Is the femoral lateral condyle’s bone morphology the trochlea of the ACL?. Knee Surg Sports Traumatol Arthrosc.

[11] Gaal BT, Knapik DM, Karns MR, Salata MJ, Voos JE (2020). Contralateral anterior cruciate ligament injuries following index reconstruction in the pediatric athlete. Curr Rev Musculoskelet Med.

[12] Grassi A, Pizza N, Zambon Bertoja J, Macchiarola L, Lucidi GA, Dal Fabbro G et al (2020) Higher risk of contralateral anterior cruciate ligament (ACL) injury within 2 years after ACL reconstruction in under-18-year-old patients with steep tibial plateau slope. Knee Surg Sports Traumatol Arthrosc DOI. 10.1007/s00167-020-06195-y 10.1007/s00167-020-06195-y 32737527

[13] Grassi A, Signorelli C, Urrizola F, Macchiarola L, Raggi F, Mosca M (2019). Patients with failed anterior cruciate ligament reconstruction have an increased posterior lateral Tibial plateau slope: a case-controlled study. Arthroscopy.

[14] Hodel S, Kabelitz M, Tondelli T, Vlachopoulos L, Sutter R, Fucentese SF (2019). Introducing the lateral femoral condyle index as a risk factor for anterior cruciate ligament injury. Am J Sports Med.

[15] Hudek R, Schmutz S, Regenfelder F, Fuchs B, Koch PP (2009). Novel measurement technique of the Tibial slope on conventional MRI. Clin Orthop Relat Res.

[16] Jaecker V, Drouven S, Naendrup JH, Kanakamedala AC, Pfeiffer T, Shafizadeh S (2018). Increased medial and lateral tibial posterior slopes are independent risk factors for graft failure following ACL reconstruction. Arch Orthop Trauma Surg.

[17] Lansdown D, Ma CB (2018). The influence of Tibial and femoral bone morphology on knee kinematics in the anterior cruciate ligament injured knee. Clin Sports Med.

[18] Lansdown DA, Pedoia V, Zaid M, Amano K, Souza RB, Li X (2017). Variations in knee kinematics after ACL injury and after reconstruction are correlated with bone shape differences. Clin Orthop Relat Res.

[19] Magnussen RA, Lawrence JT, West RL, Toth AP, Taylor DC, Garrett WE (2012). Graft size and patient age are predictors of early revision after anterior cruciate ligament reconstruction with hamstring autograft. Arthroscopy.

[20] Morgan MD, Salmon LJ, Waller A, Roe JP, Pinczewski LA (2016). Fifteen-year survival of endoscopic anterior cruciate ligament reconstruction in patients aged 18 years and younger. Am J Sports Med.

[21] Mouton C, Moksnes H, Janssen R, Fink C, Zaffagnini S, Monllau JC (2021). Preliminary experience of an international orthopaedic registry: the ESSKA Paediatric anterior cruciate ligament initiative (PAMI) registry. J Exp Orthop.

[22] Pękala Ł, Podgórski M, Shukla A, Winnicka M, Biernacka K, Grzelak P (2019). Do variants of the intercondylar notch predispose children to the injury of the anterior cruciate ligament?. Clin Anat.

[23] Pfeifer CE, Beattie PF, Sacko RS, Hand A (2018). Risk factors associated with non-contact anterior cruciate ligament injury: a systematic review. Int J Sports Phys Ther.

[24] Pfeiffer TR, Burnham JM, Hughes JD, Kanakamedala AC, Herbst E, Popchak A (2018). An increased lateral femoral condyle ratio is a risk factor for anterior cruciate ligament injury. J Bone Joint Surg Am.

[25] Pfeiffer TR, Burnham JM, Kanakamedala AC, Hughes JD, Zlotnicki J, Popchak A (2019). Distal femur morphology affects rotatory knee instability in patients with anterior cruciate ligament ruptures. Knee Surg Sports Traumatol Arthrosc.

[26] Salmon LJ, Heath E, Akrawi H, Roe JP, Linklater J, Pinczewski LA (2018). 20-year outcomes of anterior cruciate ligament reconstruction with hamstring tendon autograft: the catastrophic effect of age and posterior Tibial slope. Am J Sports Med.

[27] Sauer S, English R, Clatworthy M (2018). The ratio of Tibial slope and meniscal bone angle for the prediction of ACL reconstruction failure risk. Surg J (N Y).

[28] Shaw KA, Dunoski B, Mardis N, Pacicca D (2015). Knee morphometric risk factors for acute anterior cruciate ligament injury in skeletally immature patients. J Child Orthop.

[29] Shelbourne KD, Gray T, Haro M (2009). Incidence of subsequent injury to either knee within 5 years after anterior cruciate ligament reconstruction with patellar tendon autograft. Am J Sports Med.

[30] Sturnick DR, Vacek PM, DeSarno MJ, Gardner-Morse MG, Tourville TW, Slauterbeck JR (2018). Combined anatomic factors predicting risk of anterior cruciate ligament injury for males and females. Am J Sports Med.

[31] Vasta S, Andrade R, Pereira R, Bastos R, Battaglia AG, Papalia R (2018). Bone morphology and morphometry of the lateral femoral condyle is a risk factor for ACL injury. Knee Surg Sports Traumatol Arthrosc.

[32] Vyas S, van Eck CF, Vyas N, Fu FH, Otsuka NY (2011). Increased medial tibial slope in teenage pediatric population with open physes and anterior cruciate ligament injuries. Knee Surg Sports Traumatol Arthrosc.

[33] Webster KE, Feller JA (2016). Exploring the high reinjury rate in younger patients undergoing anterior cruciate ligament reconstruction. Am J Sports Med.

[34] Werner BC, Yang S, Looney AM, Gwathmey FW (2016). Trends in pediatric and adolescent anterior cruciate ligament injury and reconstruction. J Pediatr Orthop.

